# Case report: Total arch replacement with a frozen elephant trunk utilizing open hybrid in-situ fenestration technique for thoracic aortic arch aneurysm

**DOI:** 10.3389/fsurg.2023.1224013

**Published:** 2023-07-19

**Authors:** Yusuke Takei, I-Hui Wu, Chih-Yang Chan, Nai-Hsin Chi

**Affiliations:** ^1^ Department of Surgery, National Taiwan University Hospital and National Taiwan University College of Medicine, Taipei, Taiwan; ^2^ Department of Cardiac and Vascular Surgery, Dokkyo Medical University School of Medicine Graduate School of Medicine, Tochigi, Japan

**Keywords:** total arch replacement, frozen elephant trunk, stent graft, in-situ fenestration, thoracic aortic arch aneurysm

## Abstract

**Introduction:**

The frozen elephant trunk technique is a surgical procedure developed for concomitant repair of downstream descending thoracic aorta as a first stage operation for arch resections. Proximalization of the sutured anastomosis reduces technical difficulty of total arch replacement. In this procedure, an anastomosis is performed more proximally using a stent graft. Connect the head and neck vessels are created using in-situ fenestration method.

**Case presentation:**

This study presents the case of a 78-year-old woman with a large thoracic aortic arch aneurysm that was successfully treated with a modified frozen elephant trunk technique (open *in situ* fenestration). For this method, a hole was created in the neck branches (the left subclavian artery and left common carotid artery), and peripheral stent grafts were placed to simplify neck branch reconstruction. This minimized the risk of recurrent laryngeal nerve injury and bleeding and shortened the procedure time.

**Conclusion:**

The outcomes of this study showed a safe alternative total arch replacement procedure.

## 1. Introduction

Conventional total arch replacement (TAR) has a greater risk of bleeding and recurrent laryngeal nerve injury when dissecting aortic aneurysms. Additionally, the procedure, particularly the reconstruction of the cervical branches, is technically challenging. The frozen elephant trunk (FET) method was developed to address these issues (1), and it has achieved more favorable outcomes than conventional TAR (2). This method involved “proximalization,” which involves moving the anastomosis more proximally using a stent graft (3). This renders dissection maneuvers less essential; however, neck branch reconstruction is required. This report documents the application of a modification to a previously reported technique: the branched single anastomosis frozen elephant trunk repair (B-SAFER) (4), also called the open in-situ fenestration method, which removes the need for neck branch reconstruction by creating a hole in the neck branches, specifically the left subclavian artery (LSCA) and left common carotid artery (LCCA), and placing peripheral stent grafts. By avoiding the anastomosis of fragile artery walls and leaving the LSCA and arch untouched, this technique reduces the risk of recurrent laryngeal nerve injury, hemorrhage, and procedure time.

## 2. Case description

The procedure was approved by the Institutional Review Board of the National Taiwan University Hospital and was carried out following the principles embodied in the Declaration of Helsinki. Written informed consent was obtained from the patient.

**Figure 1:**
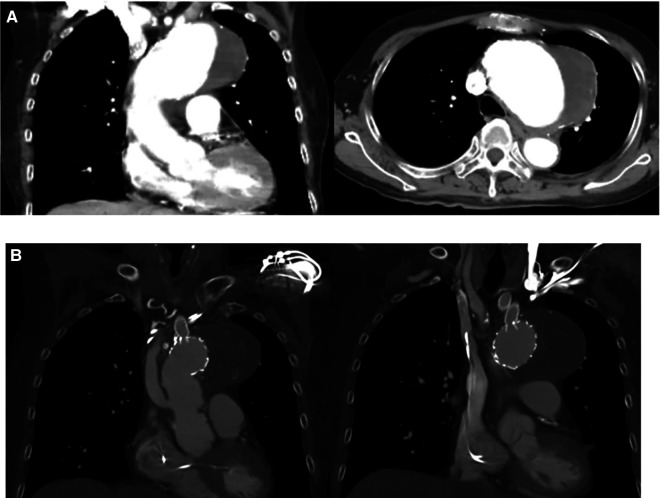
(**A**) Preoperative image. The computed tomography image shows an enlarged aortic arch with a maximal diameter of 7 cm. (**B**) Post-operative image. There was no endoleak detected, the aneurysmal sac are thrombosed and the fenestrated graft to the neck vessels are patent.

**Figure 2:**
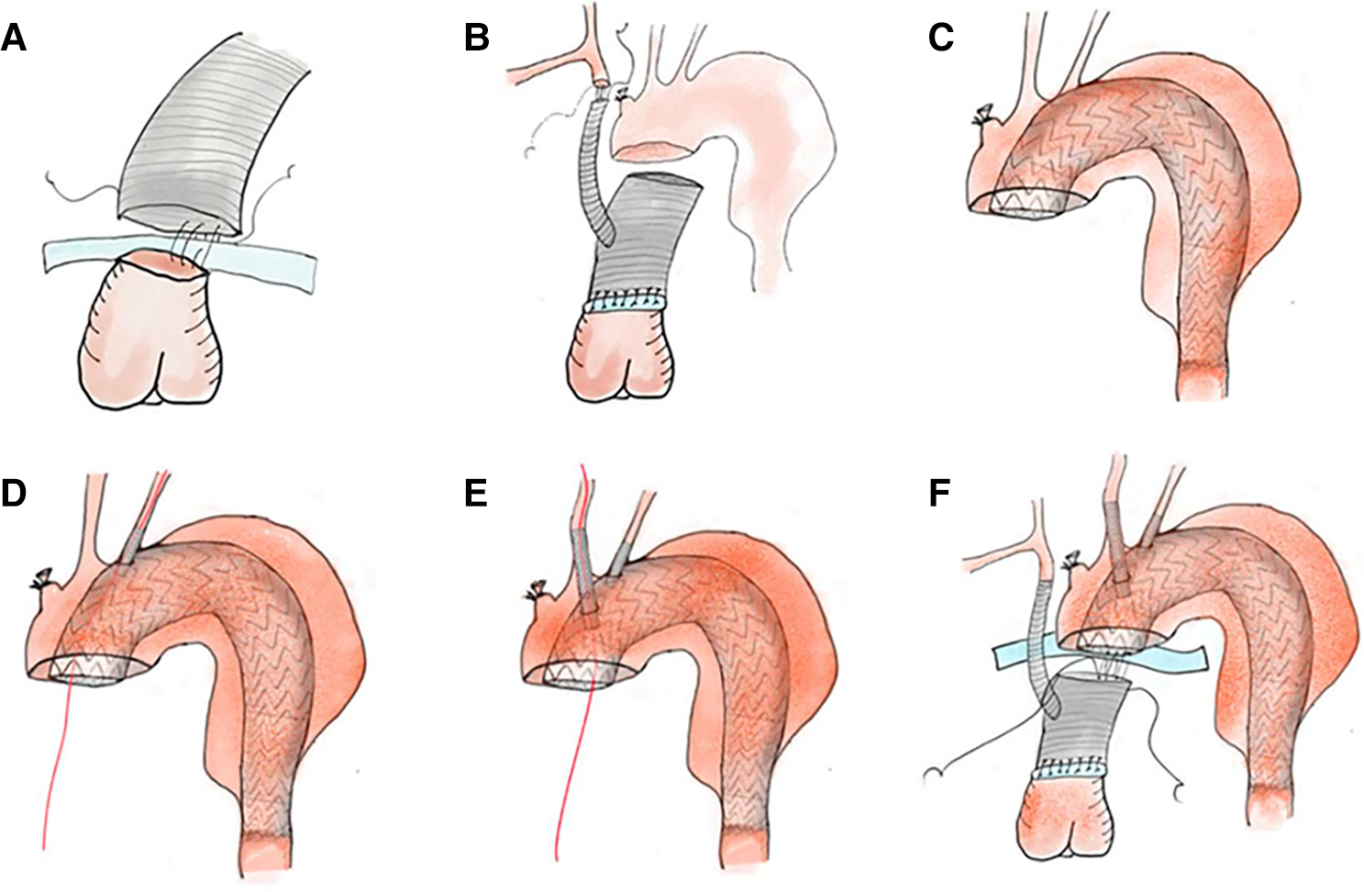
Procedures of total arch replacement with open in-situ fenestration. (**A**) Proximal anastomosis: The ascending aorta is anastomosed with a vascular graft. (**B**) BCA anastomosis: The BCA is clamped and anastomosed with the side arm of the vascular graft. (**C**) Main stent graft deployment: With direct vision, an endovascular stent graft (cTAG) is inserted into the arch. (**D,E**) Open in-situ fenestration: Both the LSCA and LCCA are fixed with covered stents by in-situ fenestration. After fenestration, the stent graft is guided by a wire for insertion and secured to both arteries. (**F**) Distal anastomosis: The proximal stent-free side of the cTAG is anastomosed with the aortic wall and distal end of the vascular graft. BCA, brachiocephalic artery; LSCA, left subclavian artery; LCCA, left common carotid artery.

**Figure 3:**
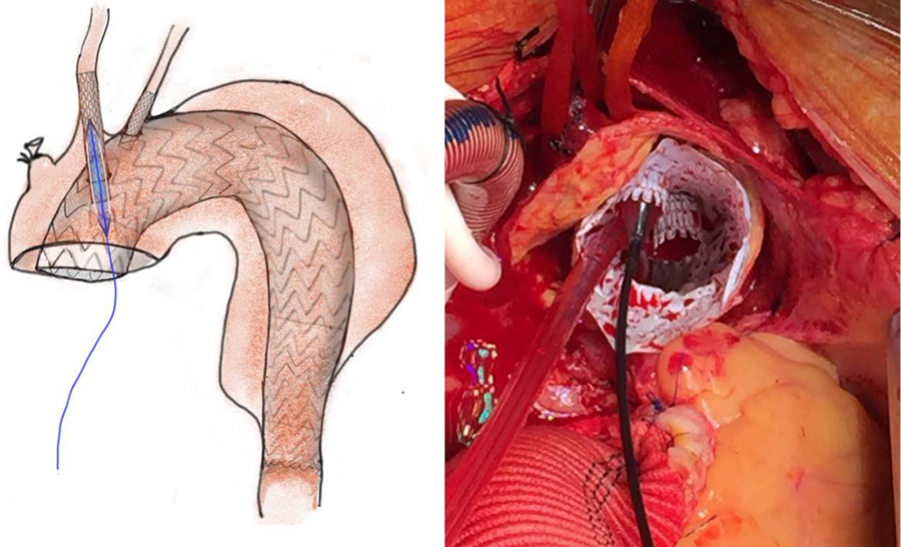
Balloon dilatation to secure the endografts with fenestration. The LCCA and LCCA endografts (VIABAHN) undergo balloon dilation to obtain fenestration and good fixation. LSCA, left subclavian artery; LCCA, left common carotid artery.

**Figure 4:**
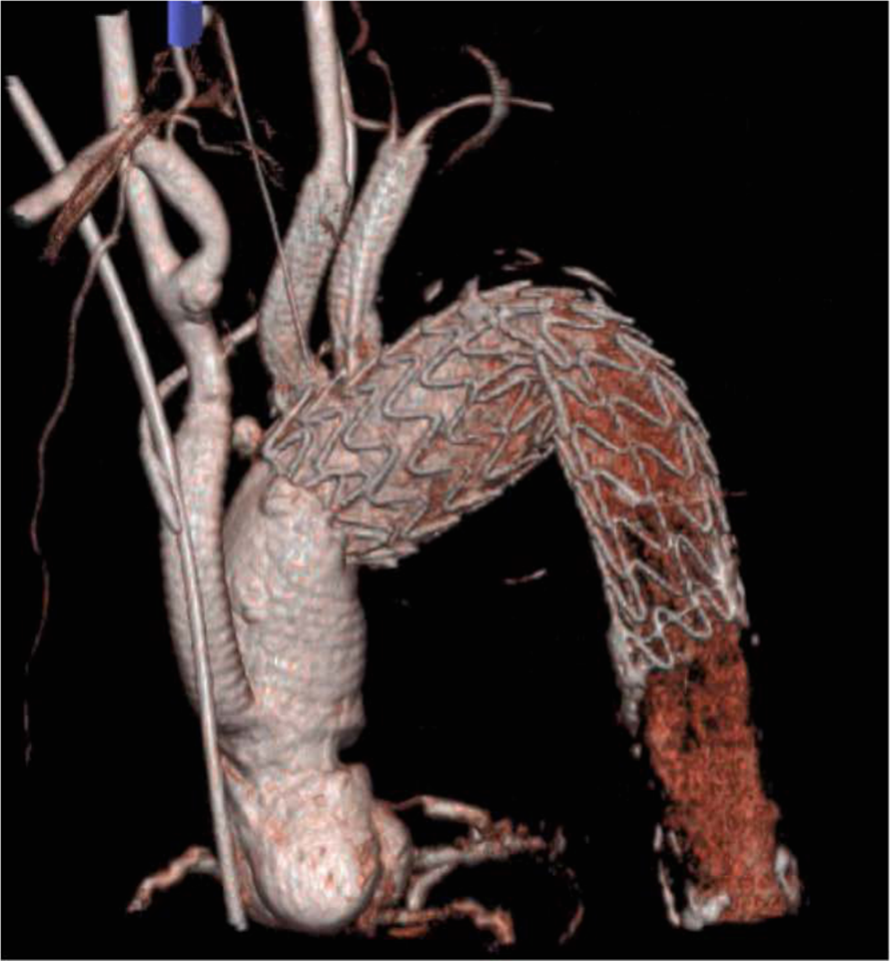
Postoperative reconstruction image. Postoperative computed tomography demonstrates adequate fixation of the main stent and two in-situ fenestration stents in the left common carotid and left subclavian arteries. The aneurysm sac was excluded from the analysis.

TAR with the FET procedure was performed using the open in-situ fenestration technique (Supplementary Video S1) on a 78-year-old woman who was diagnosed with a 7-cm thoracic aortic aneurysm incidentally detected during a medical examination (Figure 1). The patient had an unremarkable medical history. Under general anesthesia, a median sternotomy was performed, and a cardiopulmonary bypass was established via the right femoral artery and vein. An additional arterial cannula was inserted directly into the right axillary artery, and a left ventricular venting tube was placed through the right upper pulmonary vein. The ascending aorta was opened after clamping the aorta and administering antegrade cardioplegia. First, a proximal anastomosis of the ascending aorta was performed using a 4-branched knitted Dacron graft (Intergard, Getinge, Gothenburg, Sweden) with 4-0 polypropylene continuous sutures. Teflon felt strips were secured outside the suture line (Figure 2A). Second, the brachiocephalic artery (BCA) was sewn with the branch of the graft, creating a stump on the proximal side of the BCA (Figure 2B). During this process, the BCA and distal ascending aorta were clamped, while perfusion to the brain and the rest of the body was maintained. Subsequently, with a rectal temperature of 28°C, circulatory arrest was induced in the lower body, and cerebral perfusion was maintained through the right axillary cannula. Third, the aortic clamp was released, and the ascending aorta was trimmed below the BCA stump. Under a stiff guidewire, an endovascular stent graft [conformable TAG (cTAG); W. L. Gore & Associates, Inc., Newark, DE, USA] was delivered antegrade from Zone 0 to the distal arch and descending aorta (Figure 2C). To prevent endoleaks, the diameter and length of the stent graft were estimated using preoperative computed tomography angiography. After stent graft placement, the open in-situ fenestration technique was sequentially applied to the LSCA (Figure 2D) and LCCA (Figure 2E). Tiny fenestrations were created with a scalpel and enlarged using right-angle forceps from within the stent graft at a point adjacent to the orifices of the LSCA and LCCA. A 5-cm peripheral stent graft (VIABAHN, W. L. Gore & Associates, Inc.) was implanted with a 0.035-inch stiff guide wire (Radifocus, Terumo, Tokyo, Japan) and deployed through a fenestration in the LSCA. The stent graft was positioned to extend by 1 cm at the aortic side. Likewise, a second stent graft was implanted in the LCCA. Balloon dilatation was then performed to ensure patency and fixation of the branch stent graft to prevent endoleaks through the junction (Figure 3). Lastly, the proximal stent-free side of the primary stent graft was anastomosed with the aortic wall and distal end of the graft with four branches during antegrade cerebral perfusion (18°C, 10 ml/kg/min) through the cervical stent grafts (Figure 2F). The procedure lasted 5 h, and there were no intraoperative complications. Subsequent computed tomography angiography revealed a good arch morphology without endoleaks (Figures 1B and 4). The size of the aortic aneurysm was reduced. There was no endoleak noted in the 24-months followed and the patient had a good prognosis.

## 3. Discussion

An extensive exploration of the distal aortic arch is integral when performing TAR. This is associated with an increased risk of bleeding and recurrent laryngeal nerve injury (5). To avoid these complications, especially during acute aortic dissection, the FET procedure was developed, and several techniques for “proximalization” were established. For example, anastomosis of the left axillary artery to the LSCA has been performed (1). Alternatively, a fenestration has been created in the region of the LSCA (6), with an added peripheral stent graft, so-called B-SAFER (4, 6). Moreover, a customized branched FET was developed in Europe (7). In this report, we developed an open in-situ fenestration technique that improved upon B-SAFER and omitted LCCA and LSCA reconstruction. This modified method utilized existing tools, and the FET was inserted from Zone 0. This prevented bleeding and recurrent laryngeal nerve injury. Additionally, all suture lines were located in the proximal ascending aorta, resulting in faster hemostasis. A cTAG was used because it is the only endovascular stent graft that can be deployed while keeping the proximal end visible during antegrade stent graft insertion. Although the application of in-situ fenestration in a FET method was previously reported (8), utilization of a cTAG reportedly had no issues with fenestration dilatation, stenosis, or occlusion during the remote phase of in-situ fenestration for each endovascular stent graft (9). In this case, a Viabahn was chosen as the branch device because it was made of expanded polytetrafluoroethylene, which was the same material as that of the main stent graft and thus considered to be a better fit. Furthermore, balloon dilation prevents Type III endoleaks and stent graft stenosis. However, long-term periods in cases of B-SAFER in aortic dissection, and cases of residual aortic aneurysm expansion, have been reported due to persistent distal flow into the false lumen (10), so it was critical to monitor its efficacy in our patient.

One concern with this procedure is the risk of vascular injury or stroke during wire manipulation, especially in patients with a dissected flap and atheroma around the cervical branches. However, this problem has not been encountered in practice. Preoperative computed tomography angiography should be used to confirm the characteristics of the neck vessels. Since this technique has only been performed on several patients, further studies with a larger sample size are needed to validate these results and to clarify the limitations of this technique.

## 4. Conclusion

The open hybrid in-situ fenestration technique simplified the FET procedure by modifying cervical branch reconstruction using an endovascular stent graft. This prevented aggressive dissection associated with bleeding and recurrent laryngeal nerve injury. The outcomes of this study showed a safe alternative TAR procedure.

## Data Availability

The data analyzed in this study is subject to the following licenses/restrictions: The data contains information that could compromise the privacy of the research participant. Requests to access these datasets should be directed to N-HC, chinaihsin@gmail.com.

## Supplementary material

Supplementary Video S1 Total arch replacement with open in-situ fenestration. The fenestration of the stent graft and deployment of the peripheral stent graft are described the film.
